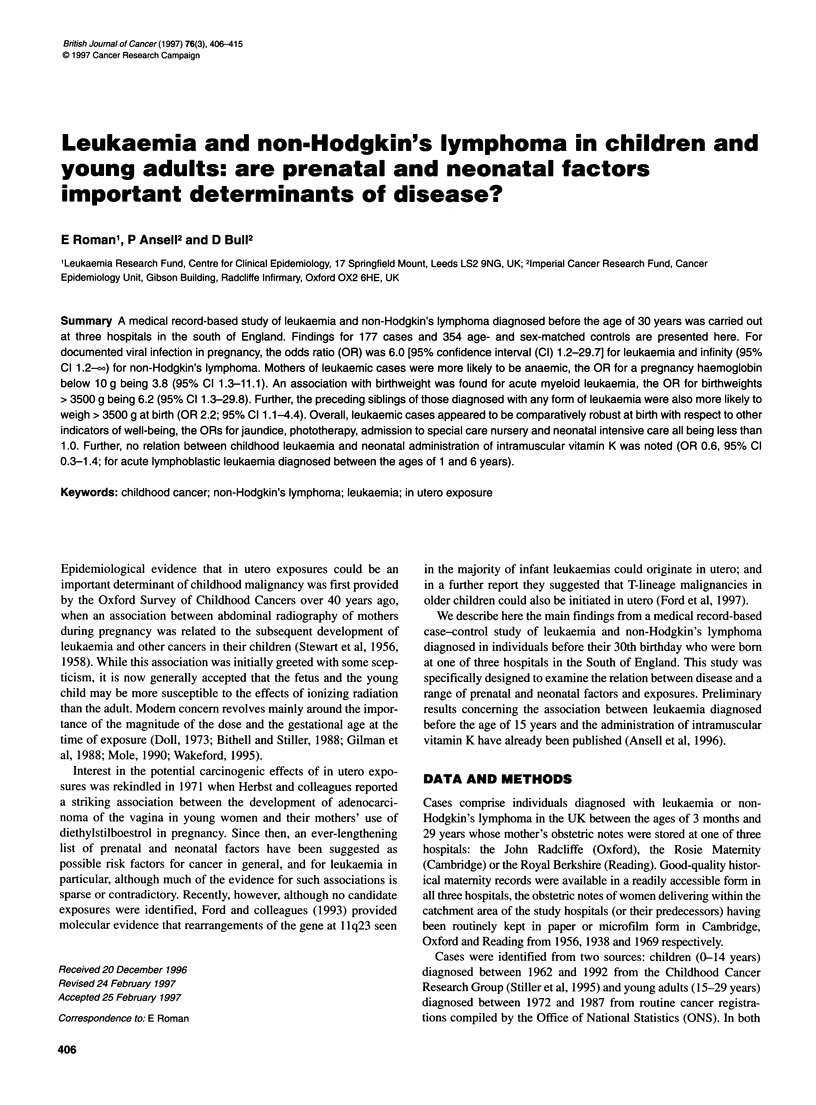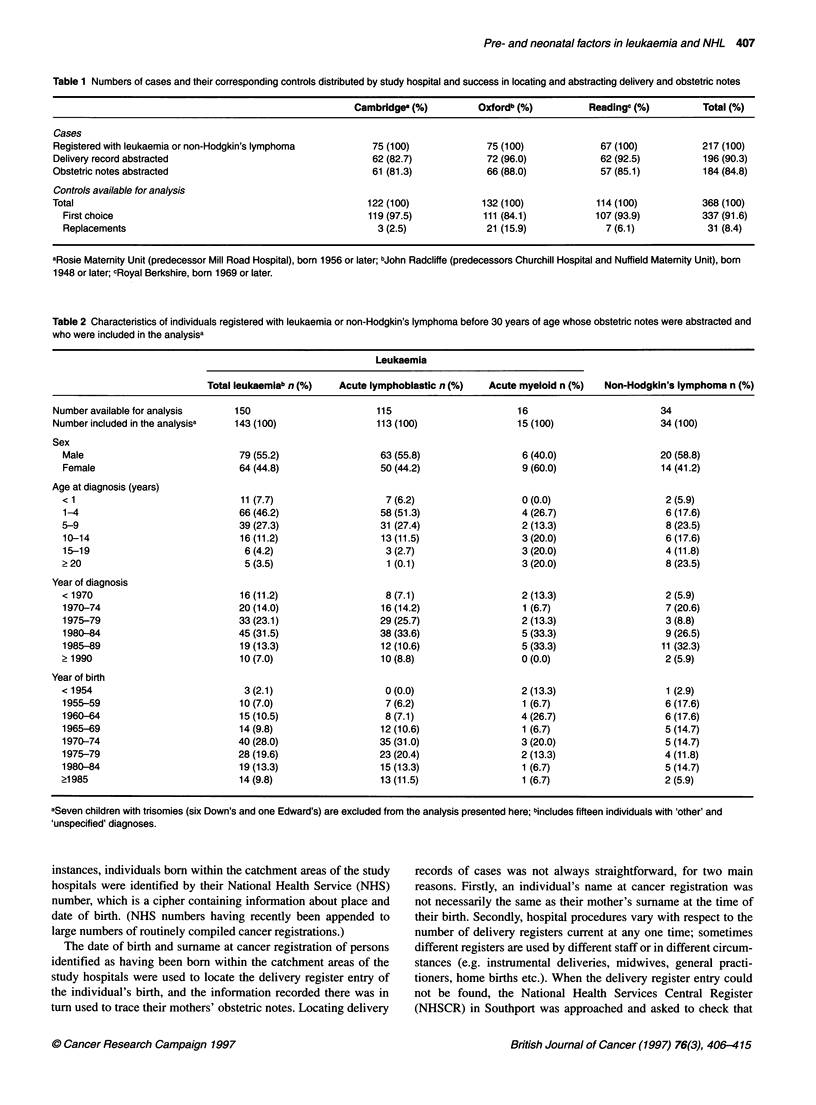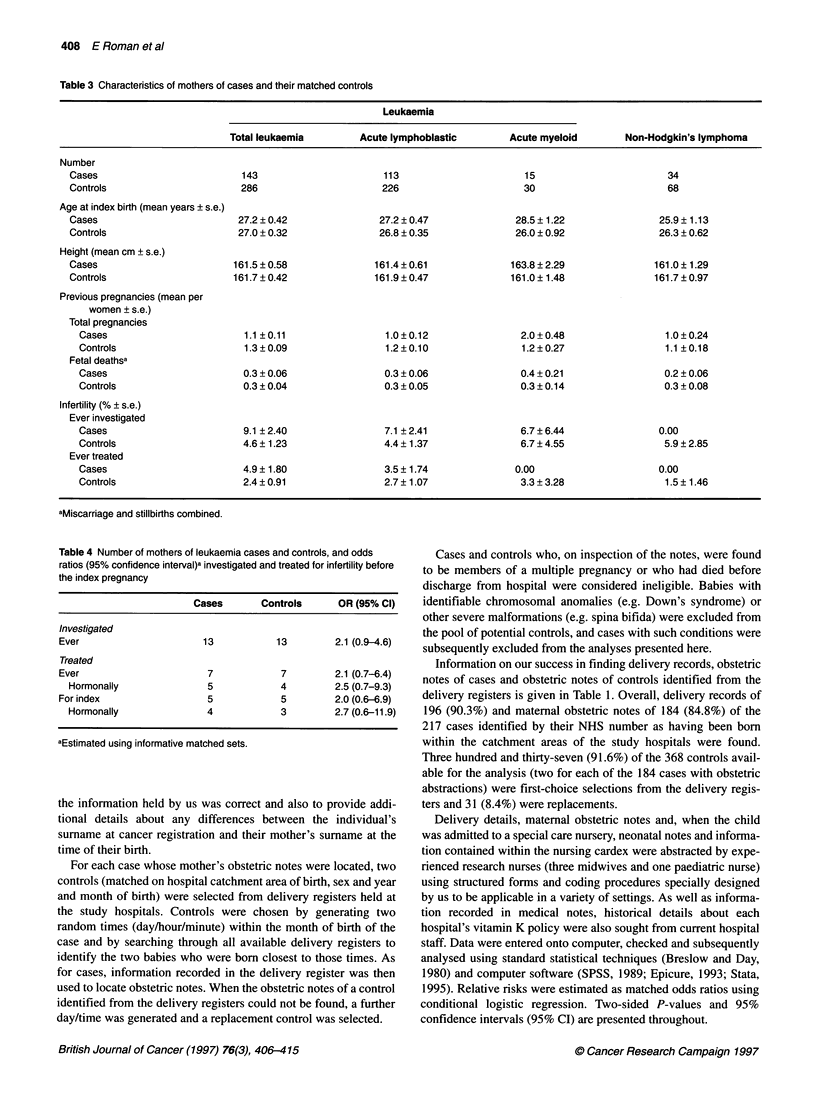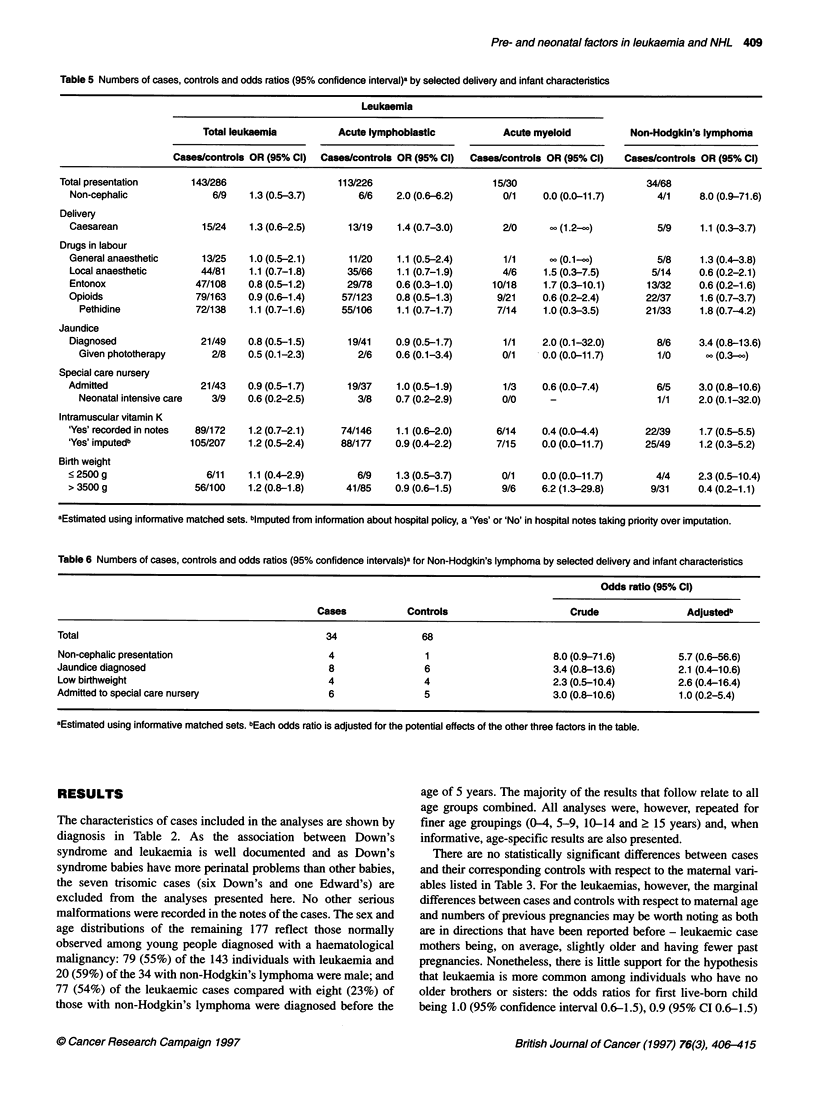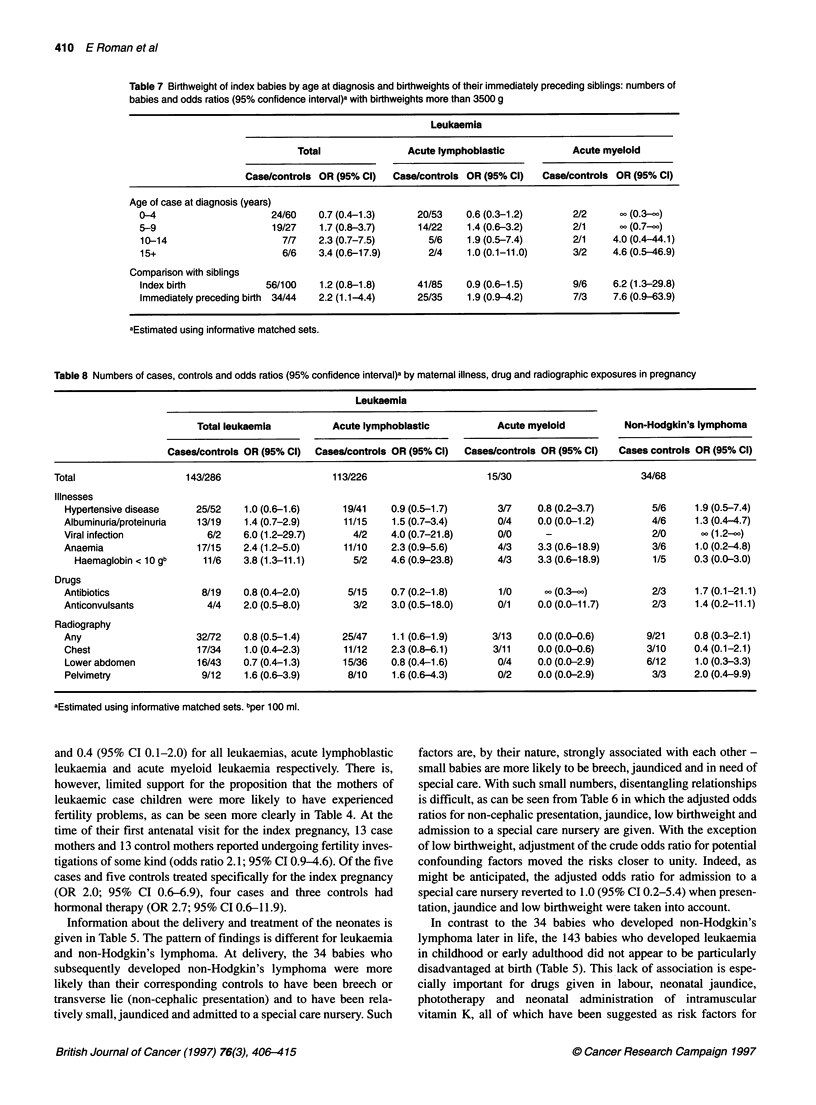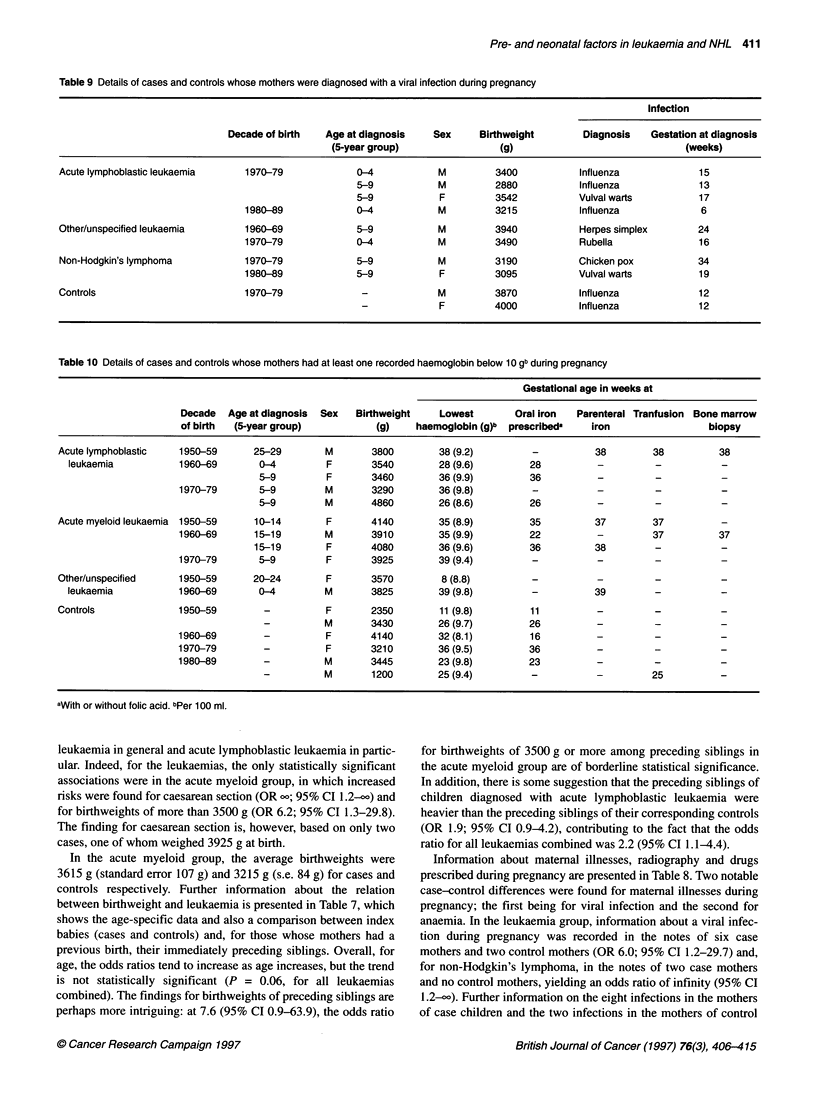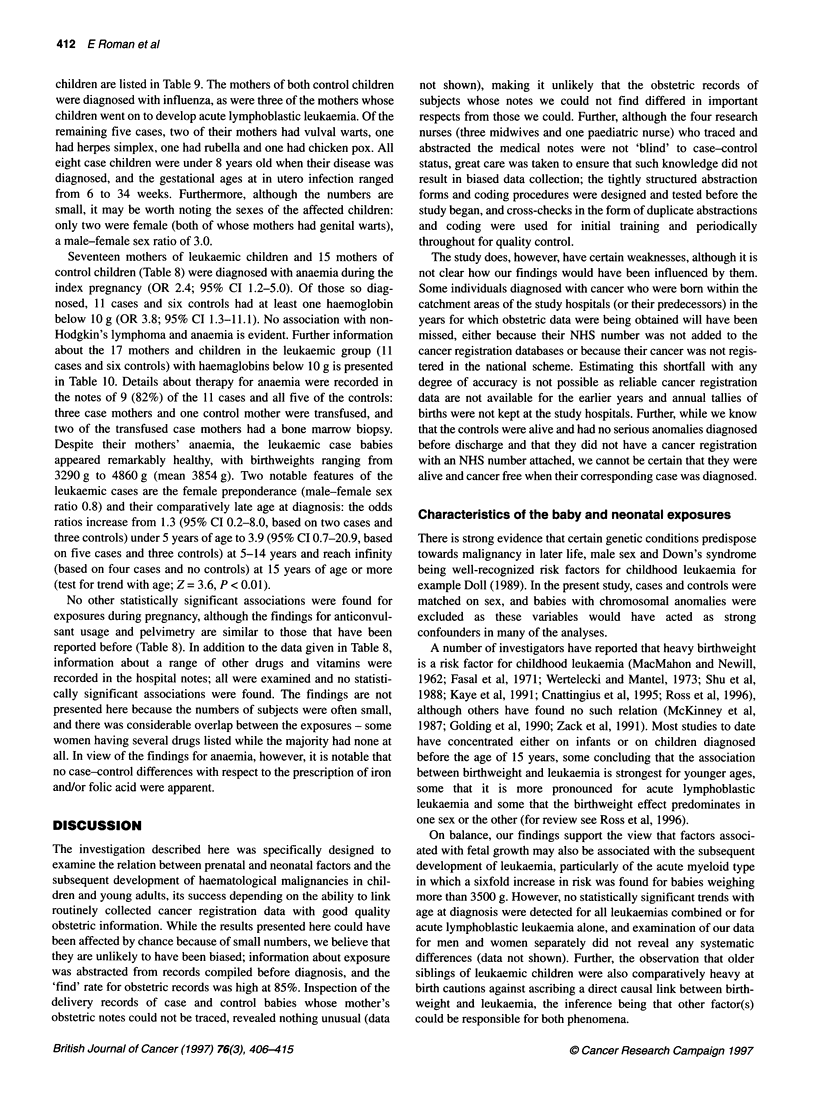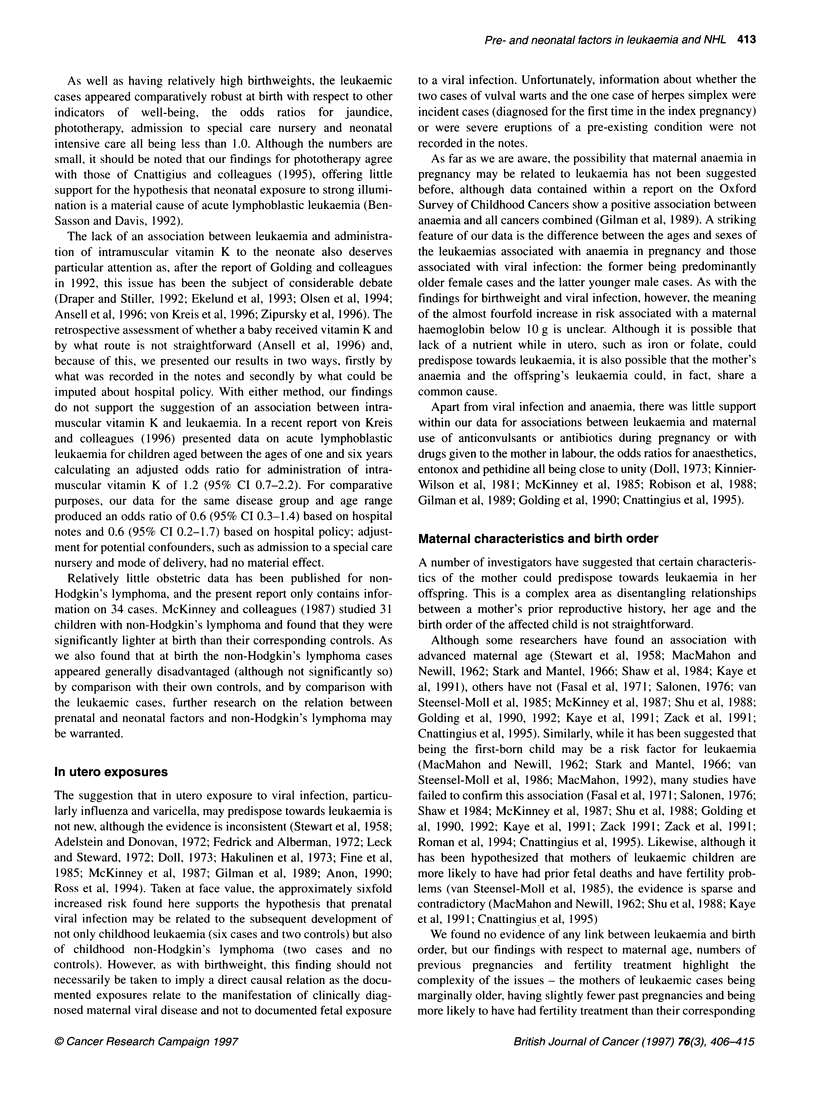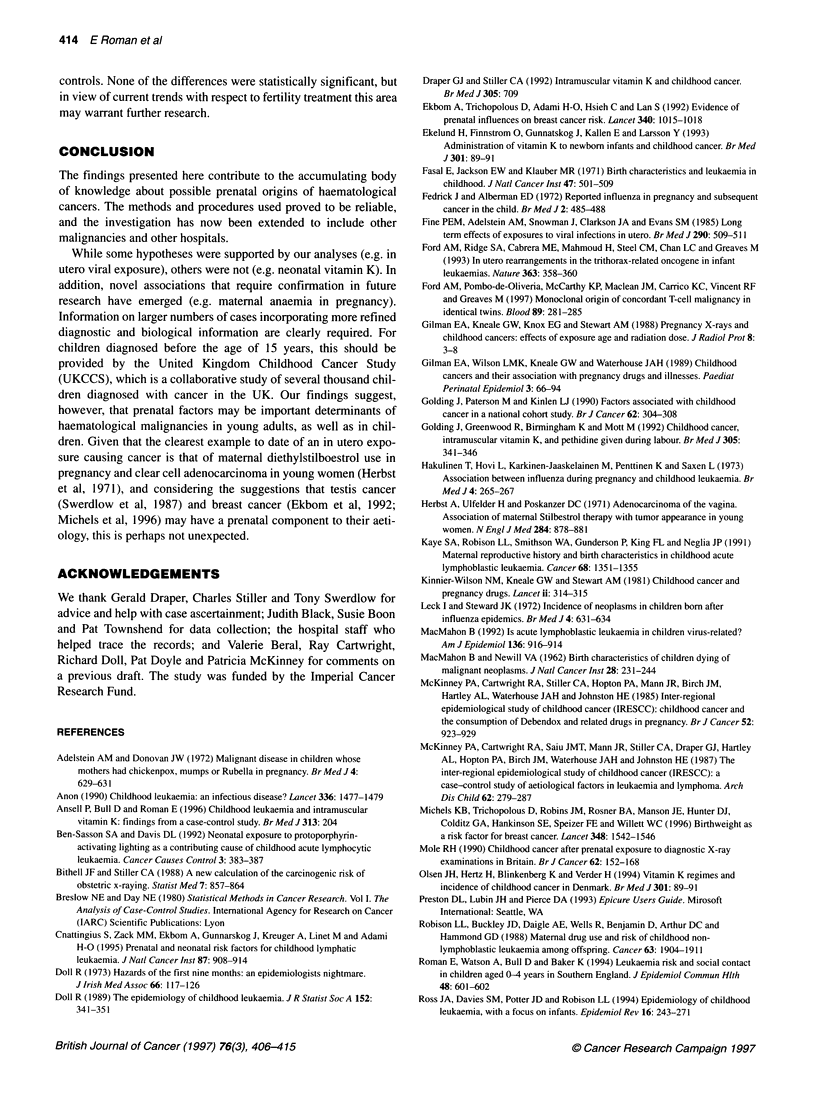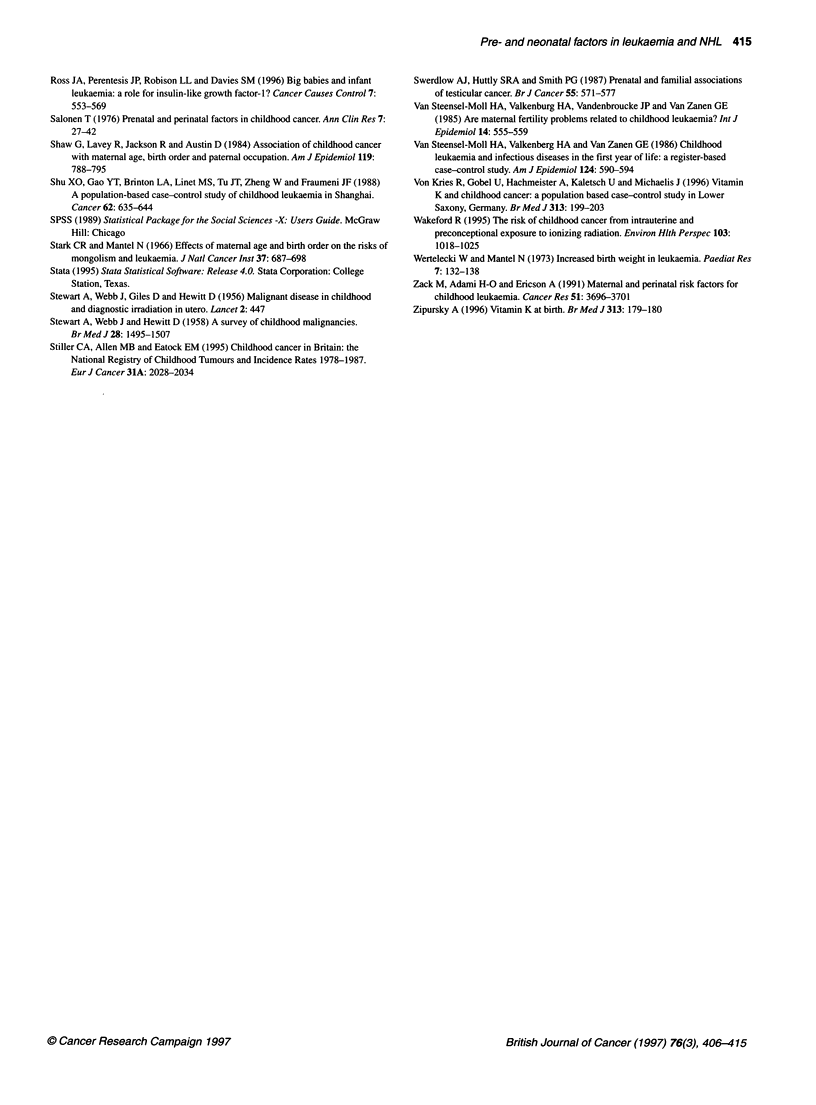# Leukaemia and non-Hodgkin's lymphoma in children and young adults: are prenatal and neonatal factors important determinants of disease?

**DOI:** 10.1038/bjc.1997.399

**Published:** 1997

**Authors:** E. Roman, P. Ansell, D. Bull

**Affiliations:** Leukaemia Research Fund, Centre for Clinical Epidemiology, Leeds, UK.

## Abstract

A medical record-based study of leukaemia and non-Hodgkin's lymphoma diagnosed before the age of 30 years was carried out at three hospitals in the south of England. Findings for 177 cases and 354 age- and sex-matched controls are presented here. For documented viral infection in pregnancy, the odds ratio (OR) was 6.0 [95% confidence interval (CI) 1.2-29.7] for leukaemia and infinity (95% CI 1.24-infinity) for non-Hodgkin's lymphoma. Mothers of leukaemic cases were more likely to be anaemic, the OR for a pregnancy haemoglobin below 10 g being 3.8 (95% CI 1.3-11.1). An association with birthweight was found for acute myeloid leukaemia, the OR for birthweights > 3500 g being 6.2 (95% CI 1.3-29.8). Further, the preceding siblings of those diagnosed with any form of leukaemia were also more likely to weigh > 3500 g at birth (OR 2.2; 95% CI 1.1-4.4). Overall, leukaemic cases appeared to be comparatively robust at birth with respect to other indicators of well-being, the ORs for jaundice, phototherapy, admission to special care nursery and neonatal intensive care all being less than 1.0. Further, no relation between childhood leukaemia and neonatal administration of intramuscular vitamin K was noted (OR 0.6, 95% CI 0.3-1.4; for acute lymphoblastic leukaemia diagnosed between the ages of 1 and 6 years).


British Journal of Cancer (1997) 76(3), 406–415
© 1997 Cancer Research Campaign

### Leukaemia and non-Hodgkin's lymphoma in children and young adults: are prenatal and neonatal factors important determinants of disease?

**E Roman[1], P Ansell[2] and D Bull[2]**

[1]Leukaemia Research Fund, Centre for Clinical Epidemiology, 17 Springfield Mount, Leeds LS2 9NG, UK; [2]Imperial Cancer Research Fund, Cancer Epidemiology Unit, Gibson Building, Radcliffe Infirmary, Oxford OX2 6HE, UK

**Summary** A medical record-based study of leukaemia and non-Hodgkin's lymphoma diagnosed before the age of 30 years was carried out at three hospitals in the south of England. Findings for 177 cases and 354 age- and sex-matched controls are presented here. For documented viral infection in pregnancy, the odds ratio (OR) was 6.0 [95% confidence interval (CI) 1.2–29.7] for leukaemia and infinity (95% CI 1.2–∞) for non-Hodgkin's lymphoma. Mothers of leukaemic cases were more likely to be anaemic, the OR for a pregnancy haemoglobin below 10 g being 3.8 (95% CI 1.3–11.1). An association with birthweight was found for acute myeloid leukaemia, the OR for birthweights > 3500 g being 6.2 (95% CI 1.3–29.8). Further, the preceding siblings of those diagnosed with any form of leukaemia were also more likely to weigh > 3500 g at birth (OR 2.2; 95% CI 1.1–4.4). Overall, leukaemic cases appeared to be comparatively robust at birth with respect to other indicators of well-being, the ORs for jaundice, phototherapy, admission to special care nursery and neonatal intensive care all being less than 1.0. Further, no relation between childhood leukaemia and neonatal administration of intramuscular vitamin K was noted (OR 0.6, 95% CI 0.3–1.4; for acute lymphoblastic leukaemia diagnosed between the ages of 1 and 6 years).

**Keywords:** childhood cancer; non-Hodgkin's lymphoma; leukaemia; in utero exposure

Epidemiological evidence that in utero exposures could be an important determinant of childhood malignancy was first provided by the Oxford Survey of Childhood Cancers over 40 years ago, when an association between abdominal radiography of mothers during pregnancy was related to the subsequent development of leukaemia and other cancers in their children (Stewart et al, 1956, 1958). While this association was initially greeted with some scepticism, it is now generally accepted that the fetus and the young child may be more susceptible to the effects of ionizing radiation than the adult. Modern concern revolves mainly around the importance of the magnitude of the dose and the gestational age at the time of exposure (Doll, 1973; Bithell and Stiller, 1988; Gilman et al, 1988; Mole, 1990; Wakeford, 1995).

Interest in the potential carcinogenic effects of in utero exposures was rekindled in 1971 when Herbst and colleagues reported a striking association between the development of adenocarcinoma of the vagina in young women and their mothers' use of diethylstilboestrol in pregnancy. Since then, an ever-lengthening list of prenatal and neonatal factors have been suggested as possible risk factors for cancer in general, and for leukaemia in particular, although much of the evidence for such associations is sparse or contradictory. Recently, however, although no candidate exposures were identified, Ford and colleagues (1993) provided molecular evidence that rearrangements of the gene at 11q23 seen in the majority of infant leukaemias could originate in utero; and in a further report they suggested that T-lineage malignancies in older children could also be initiated in utero (Ford et al, 1997).

We describe here the main findings from a medical record-based case–control study of leukaemia and non-Hodgkin's lymphoma diagnosed in individuals before their 30th birthday who were born at one of three hospitals in the South of England. This study was specifically designed to examine the relation between disease and a range of prenatal and neonatal factors and exposures. Preliminary results concerning the association between leukaemia diagnosed before the age of 15 years and the administration of intramuscular vitamin K have already been published (Ansell et al, 1996).

### DATA AND METHODS

Cases comprise individuals diagnosed with leukaemia or non-Hodgkin's lymphoma in the UK between the ages of 3 months and 29 years whose mother's obstetric notes were stored at one of three hospitals: the John Radcliffe (Oxford), the Rosie Maternity (Cambridge) or the Royal Berkshire (Reading). Good-quality historical maternity records were available in a readily accessible form in all three hospitals, the obstetric notes of women delivering within the catchment area of the study hospitals (or their predecessors) having been routinely kept in paper or microfilm form in Cambridge, Oxford and Reading from 1956, 1938 and 1969 respectively.

Cases were identified from two sources: children (0–14 years) diagnosed between 1962 and 1992 from the Childhood Cancer Research Group (Stiller et al, 1995) and young adults (15–29 years) diagnosed between 1972 and 1987 from routine cancer registrations compiled by the Office of National Statistics (ONS). In both

Received 20 December 1996
Revised 24 February 1997
Accepted 25 February 1997

Correspondence to: E Roman

**Table 1** Numbers of cases and their corresponding controls distributed by study hospital and success in locating and abstracting delivery and obstetric notes

| | Cambridge[a] (%) | Oxford[b] (%) | Reading[c] (%) | Total (%) |
|---|---|---|---|---|
| *Cases* | | | | |
| Registered with leukaemia or non-Hodgkin's lymphoma | 75 (100) | 75 (100) | 67 (100) | 217 (100) |
| Delivery record abstracted | 62 (82.7) | 72 (96.0) | 62 (92.5) | 196 (90.3) |
| Obstetric notes abstracted | 61 (81.3) | 66 (88.0) | 57 (85.1) | 184 (84.8) |
| *Controls available for analysis* | | | | |
| Total | 122 (100) | 132 (100) | 114 (100) | 368 (100) |
| First choice | 119 (97.5) | 111 (84.1) | 107 (93.9) | 337 (91.6) |
| Replacements | 3 (2.5) | 21 (15.9) | 7 (6.1) | 31 (8.4) |

[a]Rosie Maternity Unit (predecessor Mill Road Hospital), born 1956 or later; [b]John Radcliffe (predecessors Churchill Hospital and Nuffield Maternity Unit), born 1948 or later; [c]Royal Berkshire, born 1969 or later.

**Table 2** Characteristics of individuals registered with leukaemia or non-Hodgkin's lymphoma before 30 years of age whose obstetric notes were abstracted and who were included in the analysis[a]

| | Leukaemia | | | |
|---|---|---|---|---|
| | Total leukaemia[b] *n* (%) | Acute lymphoblastic *n* (%) | Acute myeloid n (%) | Non-Hodgkin's lymphoma n (%) |
| Number available for analysis | 150 | 115 | 16 | 34 |
| Number included in the analysis[a] | 143 (100) | 113 (100) | 15 (100) | 34 (100) |
| Sex | | | | |
| Male | 79 (55.2) | 63 (55.8) | 6 (40.0) | 20 (58.8) |
| Female | 64 (44.8) | 50 (44.2) | 9 (60.0) | 14 (41.2) |
| Age at diagnosis (years) | | | | |
| < 1 | 11 (7.7) | 7 (6.2) | 0 (0.0) | 2 (5.9) |
| 1–4 | 66 (46.2) | 58 (51.3) | 4 (26.7) | 6 (17.6) |
| 5–9 | 39 (27.3) | 31 (27.4) | 2 (13.3) | 8 (23.5) |
| 10–14 | 16 (11.2) | 13 (11.5) | 3 (20.0) | 6 (17.6) |
| 15–19 | 6 (4.2) | 3 (2.7) | 3 (20.0) | 4 (11.8) |
| ≥ 20 | 5 (3.5) | 1 (0.1) | 3 (20.0) | 8 (23.5) |
| Year of diagnosis | | | | |
| < 1970 | 16 (11.2) | 8 (7.1) | 2 (13.3) | 2 (5.9) |
| 1970–74 | 20 (14.0) | 16 (14.2) | 1 (6.7) | 7 (20.6) |
| 1975–79 | 33 (23.1) | 29 (25.7) | 2 (13.3) | 3 (8.8) |
| 1980–84 | 45 (31.5) | 38 (33.6) | 5 (33.3) | 9 (26.5) |
| 1985–89 | 19 (13.3) | 12 (10.6) | 5 (33.3) | 11 (32.3) |
| ≥ 1990 | 10 (7.0) | 10 (8.8) | 0 (0.0) | 2 (5.9) |
| Year of birth | | | | |
| < 1954 | 3 (2.1) | 0 (0.0) | 2 (13.3) | 1 (2.9) |
| 1955–59 | 10 (7.0) | 7 (6.2) | 1 (6.7) | 6 (17.6) |
| 1960–64 | 15 (10.5) | 8 (7.1) | 4 (26.7) | 6 (17.6) |
| 1965–69 | 14 (9.8) | 12 (10.6) | 1 (6.7) | 5 (14.7) |
| 1970–74 | 40 (28.0) | 35 (31.0) | 3 (20.0) | 5 (14.7) |
| 1975–79 | 28 (19.6) | 23 (20.4) | 2 (13.3) | 4 (11.8) |
| 1980–84 | 19 (13.3) | 15 (13.3) | 1 (6.7) | 5 (14.7) |
| ≥1985 | 14 (9.8) | 13 (11.5) | 1 (6.7) | 2 (5.9) |

[a]Seven children with trisomies (six Down's and one Edward's) are excluded from the analysis presented here; [b]includes fifteen individuals with 'other' and 'unspecified' diagnoses.

instances, individuals born within the catchment areas of the study hospitals were identified by their National Health Service (NHS) number, which is a cipher containing information about place and date of birth. (NHS numbers having recently been appended to large numbers of routinely compiled cancer registrations.)

The date of birth and surname at cancer registration of persons identified as having been born within the catchment areas of the study hospitals were used to locate the delivery register entry of the individual's birth, and the information recorded there was in turn used to trace their mothers' obstetric notes. Locating delivery records of cases was not always straightforward, for two main reasons. Firstly, an individual's name at cancer registration was not necessarily the same as their mother's surname at the time of their birth. Secondly, hospital procedures vary with respect to the number of delivery registers current at any one time; sometimes different registers are used by different staff or in different circumstances (e.g. instrumental deliveries, midwives, general practitioners, home births etc.). When the delivery register entry could not be found, the National Health Services Central Register (NHSCR) in Southport was approached and asked to check that

© Cancer Research Campaign 1997 

**Table 3** Characteristics of mothers of cases and their matched controls

| | Leukaemia | | | |
|---|---|---|---|---|
| | **Total leukaemia** | **Acute lymphoblastic** | **Acute myeloid** | **Non-Hodgkin's lymphoma** |
| Number | | | | |
| Cases | 143 | 113 | 15 | 34 |
| Controls | 286 | 226 | 30 | 68 |
| Age at index birth (mean years ± s.e.) | | | | |
| Cases | 27.2 ± 0.42 | 27.2 ± 0.47 | 28.5 ± 1.22 | 25.9 ± 1.13 |
| Controls | 27.0 ± 0.32 | 26.8 ± 0.35 | 26.0 ± 0.92 | 26.3 ± 0.62 |
| Height (mean cm ± s.e.) | | | | |
| Cases | 161.5 ± 0.58 | 161.4 ± 0.61 | 163.8 ± 2.29 | 161.0 ± 1.29 |
| Controls | 161.7 ± 0.42 | 161.9 ± 0.47 | 161.0 ± 1.48 | 161.7 ± 0.97 |
| Previous pregnancies (mean per women ± s.e.) | | | | |
| Total pregnancies | | | | |
| Cases | 1.1 ± 0.11 | 1.0 ± 0.12 | 2.0 ± 0.48 | 1.0 ± 0.24 |
| Controls | 1.3 ± 0.09 | 1.2 ± 0.10 | 1.2 ± 0.27 | 1.1 ± 0.18 |
| Fetal deaths[a] | | | | |
| Cases | 0.3 ± 0.06 | 0.3 ± 0.06 | 0.4 ± 0.21 | 0.2 ± 0.06 |
| Controls | 0.3 ± 0.04 | 0.3 ± 0.05 | 0.3 ± 0.14 | 0.3 ± 0.08 |
| Infertility (% ± s.e.) | | | | |
| Ever investigated | | | | |
| Cases | 9.1 ± 2.40 | 7.1 ± 2.41 | 6.7 ± 6.44 | 0.00 |
| Controls | 4.6 ± 1.23 | 4.4 ± 1.37 | 6.7 ± 4.55 | 5.9 ± 2.85 |
| Ever treated | | | | |
| Cases | 4.9 ± 1.80 | 3.5 ± 1.74 | 0.00 | 0.00 |
| Controls | 2.4 ± 0.91 | 2.7 ± 1.07 | 3.3 ± 3.28 | 1.5 ± 1.46 |

[a]Miscarriage and stillbirths combined.

**Table 4** Number of mothers of leukaemia cases and controls, and odds ratios (95% confidence interval)[a] investigated and treated for infertility before the index pregnancy

| | **Cases** | **Controls** | **OR (95% CI)** |
|---|---|---|---|
| *Investigated* | | | |
| Ever | 13 | 13 | 2.1 (0.9–4.6) |
| *Treated* | | | |
| Ever | 7 | 7 | 2.1 (0.7–6.4) |
| Hormonally | 5 | 4 | 2.5 (0.7–9.3) |
| For index | 5 | 5 | 2.0 (0.6–6.9) |
| Hormonally | 4 | 3 | 2.7 (0.6–11.9) |

[a]Estimated using informative matched sets.

the information held by us was correct and also to provide additional details about any differences between the individual's surname at cancer registration and their mother's surname at the time of their birth.

For each case whose mother's obstetric notes were located, two controls (matched on hospital catchment area of birth, sex and year and month of birth) were selected from delivery registers held at the study hospitals. Controls were chosen by generating two random times (day/hour/minute) within the month of birth of the case and by searching through all available delivery registers to identify the two babies who were born closest to those times. As for cases, information recorded in the delivery register was then used to locate obstetric notes. When the obstetric notes of a control identified from the delivery registers could not be found, a further day/time was generated and a replacement control was selected.

Cases and controls who, on inspection of the notes, were found to be members of a multiple pregnancy or who had died before discharge from hospital were considered ineligible. Babies with identifiable chromosomal anomalies (e.g. Down's syndrome) or other severe malformations (e.g. spina bifida) were excluded from the pool of potential controls, and cases with such conditions were subsequently excluded from the analyses presented here.

Information on our success in finding delivery records, obstetric notes of cases and obstetric notes of controls identified from the delivery registers is given in Table 1. Overall, delivery records of 196 (90.3%) and maternal obstetric notes of 184 (84.8%) of the 217 cases identified by their NHS number as having been born within the catchment areas of the study hospitals were found. Three hundred and thirty-seven (91.6%) of the 368 controls available for the analysis (two for each of the 184 cases with obstetric abstractions) were first-choice selections from the delivery registers and 31 (8.4%) were replacements.

Delivery details, maternal obstetric notes and, when the child was admitted to a special care nursery, neonatal notes and information contained within the nursing cardex were abstracted by experienced research nurses (three midwives and one paediatric nurse) using structured forms and coding procedures specially designed by us to be applicable in a variety of settings. As well as information recorded in medical notes, historical details about each hospital's vitamin K policy were also sought from current hospital staff. Data were entered onto computer, checked and subsequently analysed using standard statistical techniques (Breslow and Day, 1980) and computer software (SPSS, 1989; Epicure, 1993; Stata, 1995). Relative risks were estimated as matched odds ratios using conditional logistic regression. Two-sided *P*-values and 95% confidence intervals (95% CI) are presented throughout.

*© Cancer Research Campaign 1997*

**Table 5** Numbers of cases, controls and odds ratios (95% confidence interval)[a] by selected delivery and infant characteristics

| | Leukaemia | | | | | | Non-Hodgkin's lymphoma | |
|---|---|---|---|---|---|---|---|---|
| | Total leukaemia | | Acute lymphoblastic | | Acute myeloid | | | |
| | Cases/controls | OR (95% CI) | Cases/controls | OR (95% CI) | Cases/controls | OR (95% CI) | Cases/controls | OR (95% CI) |
| Total presentation | 143/286 | | 113/226 | | 15/30 | | 34/68 | |
| Non-cephalic | 6/9 | 1.3 (0.5–3.7) | 6/6 | 2.0 (0.6–6.2) | 0/1 | 0.0 (0.0–11.7) | 4/1 | 8.0 (0.9–71.6) |
| Delivery | | | | | | | | |
| Caesarean | 15/24 | 1.3 (0.6–2.5) | 13/19 | 1.4 (0.7–3.0) | 2/0 | ∞ (1.2–∞) | 5/9 | 1.1 (0.3–3.7) |
| Drugs in labour | | | | | | | | |
| General anaesthetic | 13/25 | 1.0 (0.5–2.1) | 11/20 | 1.1 (0.5–2.4) | 1/1 | ∞ (0.1–∞) | 5/8 | 1.3 (0.4–3.8) |
| Local anaesthetic | 44/81 | 1.1 (0.7–1.8) | 35/66 | 1.1 (0.7–1.9) | 4/6 | 1.5 (0.3–7.5) | 5/14 | 0.6 (0.2–2.1) |
| Entonox | 47/108 | 0.8 (0.5–1.2) | 29/78 | 0.6 (0.3–1.0) | 10/18 | 1.7 (0.3–10.1) | 13/32 | 0.6 (0.2–1.6) |
| Opioids | 79/163 | 0.9 (0.6–1.4) | 57/123 | 0.8 (0.5–1.3) | 9/21 | 0.6 (0.2–2.4) | 22/37 | 1.6 (0.7–3.7) |
| Pethidine | 72/138 | 1.1 (0.7–1.6) | 55/106 | 1.1 (0.7–1.7) | 7/14 | 1.0 (0.3–3.5) | 21/33 | 1.8 (0.7–4.2) |
| Jaundice | | | | | | | | |
| Diagnosed | 21/49 | 0.8 (0.5–1.5) | 19/41 | 0.9 (0.5–1.7) | 1/1 | 2.0 (0.1–32.0) | 8/6 | 3.4 (0.8–13.6) |
| Given phototherapy | 2/8 | 0.5 (0.1–2.3) | 2/6 | 0.6 (0.1–3.4) | 0/1 | 0.0 (0.0–11.7) | 1/0 | ∞ (0.3–∞) |
| Special care nursery | | | | | | | | |
| Admitted | 21/43 | 0.9 (0.5–1.7) | 19/37 | 1.0 (0.5–1.9) | 1/3 | 0.6 (0.0–7.4) | 6/5 | 3.0 (0.8–10.6) |
| Neonatal intensive care | 3/9 | 0.6 (0.2–2.5) | 3/8 | 0.7 (0.2–2.9) | 0/0 | – | 1/1 | 2.0 (0.1–32.0) |
| Intramuscular vitamin K | | | | | | | | |
| 'Yes' recorded in notes | 89/172 | 1.2 (0.7–2.1) | 74/146 | 1.1 (0.6–2.0) | 6/14 | 0.4 (0.0–4.4) | 22/39 | 1.7 (0.5–5.5) |
| 'Yes' imputed[b] | 105/207 | 1.2 (0.5–2.4) | 88/177 | 0.9 (0.4–2.2) | 7/15 | 0.0 (0.0–11.7) | 25/49 | 1.2 (0.3–5.2) |
| Birth weight | | | | | | | | |
| ≤ 2500 g | 6/11 | 1.1 (0.4–2.9) | 6/9 | 1.3 (0.5–3.7) | 0/1 | 0.0 (0.0–11.7) | 4/4 | 2.3 (0.5–10.4) |
| > 3500 g | 56/100 | 1.2 (0.8–1.8) | 41/85 | 0.9 (0.6–1.5) | 9/6 | 6.2 (1.3–29.8) | 9/31 | 0.4 (0.2–1.1) |

[a]Estimated using informative matched sets. [b]Imputed from information about hospital policy, a 'Yes' or 'No' in hospital notes taking priority over imputation.

**Table 6** Numbers of cases, controls and odds ratios (95% confidence intervals)[a] for Non-Hodgkin's lymphoma by selected delivery and infant characteristics

| | | | Odds ratio (95% CI) | |
|---|---|---|---|---|
| | Cases | Controls | Crude | Adjusted[b] |
| Total | 34 | 68 | | |
| Non-cephalic presentation | 4 | 1 | 8.0 (0.9–71.6) | 5.7 (0.6–56.6) |
| Jaundice diagnosed | 8 | 6 | 3.4 (0.8–13.6) | 2.1 (0.4–10.6) |
| Low birthweight | 4 | 4 | 2.3 (0.5–10.4) | 2.6 (0.4–16.4) |
| Admitted to special care nursery | 6 | 5 | 3.0 (0.8–10.6) | 1.0 (0.2–5.4) |

[a]Estimated using informative matched sets. [b]Each odds ratio is adjusted for the potential effects of the other three factors in the table.

### RESULTS

The characteristics of cases included in the analyses are shown by diagnosis in Table 2. As the association between Down's syndrome and leukaemia is well documented and as Down's syndrome babies have more perinatal problems than other babies, the seven trisomic cases (six Down's and one Edward's) are excluded from the analyses presented here. No other serious malformations were recorded in the notes of the cases. The sex and age distributions of the remaining 177 reflect those normally observed among young people diagnosed with a haematological malignancy: 79 (55%) of the 143 individuals with leukaemia and 20 (59%) of the 34 with non-Hodgkin's lymphoma were male; and 77 (54%) of the leukaemic cases compared with eight (23%) of those with non-Hodgkin's lymphoma were diagnosed before the age of 5 years. The majority of the results that follow relate to all age groups combined. All analyses were, however, repeated for finer age groupings (0–4, 5–9, 10–14 and ≥ 15 years) and, when informative, age-specific results are also presented.

There are no statistically significant differences between cases and their corresponding controls with respect to the maternal variables listed in Table 3. For the leukaemias, however, the marginal differences between cases and controls with respect to maternal age and numbers of previous pregnancies may be worth noting as both are in directions that have been reported before – leukaemic case mothers being, on average, slightly older and having fewer past pregnancies. Nonetheless, there is little support for the hypothesis that leukaemia is more common among individuals who have no older brothers or sisters: the odds ratios for first live-born child being 1.0 (95% confidence interval 0.6–1.5), 0.9 (95% CI 0.6–1.5)

*© Cancer Research Campaign 1997*

**Table 7** Birthweight of index babies by age at diagnosis and birthweights of their immediately preceding siblings: numbers of babies and odds ratios (95% confidence interval)[a] with birthweights more than 3500 g

| | Leukaemia | | | | | |
|---|---|---|---|---|---|---|
| | Total | | Acute lymphoblastic | | Acute myeloid | |
| | Case/controls | OR (95% CI) | Case/controls | OR (95% CI) | Case/controls | OR (95% CI) |
| Age of case at diagnosis (years) | | | | | | |
| 0–4 | 24/60 | 0.7 (0.4–1.3) | 20/53 | 0.6 (0.3–1.2) | 2/2 | ∞ (0.3–∞) |
| 5–9 | 19/27 | 1.7 (0.8–3.7) | 14/22 | 1.4 (0.6–3.2) | 2/1 | ∞ (0.7–∞) |
| 10–14 | 7/7 | 2.3 (0.7–7.5) | 5/6 | 1.9 (0.5–7.4) | 2/1 | 4.0 (0.4–44.1) |
| 15+ | 6/6 | 3.4 (0.6–17.9) | 2/4 | 1.0 (0.1–11.0) | 3/2 | 4.6 (0.5–46.9) |
| Comparison with siblings | | | | | | |
| Index birth | 56/100 | 1.2 (0.8–1.8) | 41/85 | 0.9 (0.6–1.5) | 9/6 | 6.2 (1.3–29.8) |
| Immediately preceding birth | 34/44 | 2.2 (1.1–4.4) | 25/35 | 1.9 (0.9–4.2) | 7/3 | 7.6 (0.9–63.9) |

[a]Estimated using informative matched sets.

**Table 8** Numbers of cases, controls and odds ratios (95% confidence interval)[a] by maternal illness, drug and radiographic exposures in pregnancy

| | Leukaemia | | | | | | | |
|---|---|---|---|---|---|---|---|---|
| | Total leukaemia | | Acute lymphoblastic | | Acute myeloid | | Non-Hodgkin's lymphoma | |
| | Cases/controls | OR (95% CI) | Cases/controls | OR (95% CI) | Cases/controls | OR (95% CI) | Cases controls | OR (95% CI) |
| Total | 143/286 | | 113/226 | | 15/30 | | 34/68 | |
| Illnesses | | | | | | | | |
| Hypertensive disease | 25/52 | 1.0 (0.6–1.6) | 19/41 | 0.9 (0.5–1.7) | 3/7 | 0.8 (0.2–3.7) | 5/6 | 1.9 (0.5–7.4) |
| Albuminuria/proteinuria | 13/19 | 1.4 (0.7–2.9) | 11/15 | 1.5 (0.7–3.4) | 0/4 | 0.0 (0.0–1.2) | 4/6 | 1.3 (0.4–4.7) |
| Viral infection | 6/2 | 6.0 (1.2–29.7) | 4/2 | 4.0 (0.7–21.8) | 0/0 | – | 2/0 | ∞ (1.2–∞) |
| Anaemia | 17/15 | 2.4 (1.2–5.0) | 11/10 | 2.3 (0.9–5.6) | 4/3 | 3.3 (0.6–18.9) | 3/6 | 1.0 (0.2–4.8) |
| Haemaglobin < 10 g[b] | 11/6 | 3.8 (1.3–11.1) | 5/2 | 4.6 (0.9–23.8) | 4/3 | 3.3 (0.6–18.9) | 1/5 | 0.3 (0.0–3.0) |
| Drugs | | | | | | | | |
| Antibiotics | 8/19 | 0.8 (0.4–2.0) | 5/15 | 0.7 (0.2–1.8) | 1/0 | ∞ (0.3–∞) | 2/3 | 1.7 (0.1–21.1) |
| Anticonvulsants | 4/4 | 2.0 (0.5–8.0) | 3/2 | 3.0 (0.5–18.0) | 0/1 | 0.0 (0.0–11.7) | 2/3 | 1.4 (0.2–11.1) |
| Radiography | | | | | | | | |
| Any | 32/72 | 0.8 (0.5–1.4) | 25/47 | 1.1 (0.6–1.9) | 3/13 | 0.0 (0.0–0.6) | 9/21 | 0.8 (0.3–2.1) |
| Chest | 17/34 | 1.0 (0.4–2.3) | 11/12 | 2.3 (0.8–6.1) | 3/11 | 0.0 (0.0–0.6) | 3/10 | 0.4 (0.1–2.1) |
| Lower abdomen | 16/43 | 0.7 (0.4–1.3) | 15/36 | 0.8 (0.4–1.6) | 0/4 | 0.0 (0.0–2.9) | 6/12 | 1.0 (0.3–3.3) |
| Pelvimetry | 9/12 | 1.6 (0.6–3.9) | 8/10 | 1.6 (0.6–4.3) | 0/2 | 0.0 (0.0–2.9) | 3/3 | 2.0 (0.4–9.9) |

[a]Estimated using informative matched sets. [b]per 100 ml.

and 0.4 (95% CI 0.1–2.0) for all leukaemias, acute lymphoblastic leukaemia and acute myeloid leukaemia respectively. There is, however, limited support for the proposition that the mothers of leukaemic case children were more likely to have experienced fertility problems, as can be seen more clearly in Table 4. At the time of their first antenatal visit for the index pregnancy, 13 case mothers and 13 control mothers reported undergoing fertility investigations of some kind (odds ratio 2.1; 95% CI 0.9–4.6). Of the five cases and five controls treated specifically for the index pregnancy (OR 2.0; 95% CI 0.6–6.9), four cases and three controls had hormonal therapy (OR 2.7; 95% CI 0.6–11.9).

Information about the delivery and treatment of the neonates is given in Table 5. The pattern of findings is different for leukaemia and non-Hodgkin's lymphoma. At delivery, the 34 babies who subsequently developed non-Hodgkin's lymphoma were more likely than their corresponding controls to have been breech or transverse lie (non-cephalic presentation) and to have been relatively small, jaundiced and admitted to a special care nursery. Such factors are, by their nature, strongly associated with each other – small babies are more likely to be breech, jaundiced and in need of special care. With such small numbers, disentangling relationships is difficult, as can be seen from Table 6 in which the adjusted odds ratios for non-cephalic presentation, jaundice, low birthweight and admission to a special care nursery are given. With the exception of low birthweight, adjustment of the crude odds ratio for potential confounding factors moved the risks closer to unity. Indeed, as might be anticipated, the adjusted odds ratio for admission to a special care nursery reverted to 1.0 (95% CI 0.2–5.4) when presentation, jaundice and low birthweight were taken into account.

In contrast to the 34 babies who developed non-Hodgkin's lymphoma later in life, the 143 babies who developed leukaemia in childhood or early adulthood did not appear to be particularly disadvantaged at birth (Table 5). This lack of association is especially important for drugs given in labour, neonatal jaundice, phototherapy and neonatal administration of intramuscular vitamin K, all of which have been suggested as risk factors for

© Cancer Research Campaign 1997

**Table 9** Details of cases and controls whose mothers were diagnosed with a viral infection during pregnancy

| | Decade of birth | Age at diagnosis (5-year group) | Sex | Birthweight (g) | Infection | |
|---|---|---|---|---|---|---|
| | | | | | Diagnosis | Gestation at diagnosis (weeks) |
| Acute lymphoblastic leukaemia | 1970–79 | 0–4 | M | 3400 | Influenza | 15 |
| | | 5–9 | M | 2880 | Influenza | 13 |
| | | 5–9 | F | 3542 | Vulval warts | 17 |
| | 1980–89 | 0–4 | M | 3215 | Influenza | 6 |
| Other/unspecified leukaemia | 1960–69 | 5–9 | M | 3940 | Herpes simplex | 24 |
| | 1970–79 | 0–4 | M | 3490 | Rubella | 16 |
| Non-Hodgkin's lymphoma | 1970–79 | 5–9 | M | 3190 | Chicken pox | 34 |
| | 1980–89 | 5–9 | F | 3095 | Vulval warts | 19 |
| Controls | 1970–79 | – | M | 3870 | Influenza | 12 |
| | | – | F | 4000 | Influenza | 12 |

**Table 10** Details of cases and controls whose mothers had at least one recorded haemoglobin below 10 g[b] during pregnancy

| | Decade of birth | Age at diagnosis (5-year group) | Sex | Birthweight (g) | Gestational age in weeks at | | | | |
|---|---|---|---|---|---|---|---|---|---|
| | | | | | Lowest haemoglobin (g)[b] | Oral iron prescribed[a] | Parenteral iron | Tranfusion | Bone marrow biopsy |
| Acute lymphoblastic leukaemia | 1950–59 | 25–29 | M | 3800 | 38 (9.2) | – | 38 | 38 | 38 |
| | 1960–69 | 0–4 | F | 3540 | 28 (9.6) | 28 | – | – | – |
| | | 5–9 | F | 3460 | 36 (9.9) | 36 | – | – | – |
| | 1970–79 | 5–9 | M | 3290 | 36 (9.8) | – | – | – | – |
| | | 5–9 | M | 4860 | 26 (8.6) | 26 | – | – | – |
| Acute myeloid leukaemia | 1950–59 | 10–14 | F | 4140 | 35 (8.9) | 35 | 37 | 37 | – |
| | 1960–69 | 15–19 | M | 3910 | 35 (9.9) | 22 | – | 37 | 37 |
| | | 15–19 | F | 4080 | 36 (9.6) | 36 | 38 | – | – |
| | 1970–79 | 5–9 | F | 3925 | 39 (9.4) | – | – | – | – |
| Other/unspecified leukaemia | 1950–59 | 20–24 | F | 3570 | 8 (8.8) | – | – | – | – |
| | 1960–69 | 0–4 | M | 3825 | 39 (9.8) | – | 39 | – | – |
| Controls | 1950–59 | – | F | 2350 | 11 (9.8) | 11 | – | – | – |
| | | – | M | 3430 | 26 (9.7) | 26 | – | – | – |
| | 1960–69 | – | F | 4140 | 32 (8.1) | 16 | – | – | – |
| | 1970–79 | – | F | 3210 | 36 (9.5) | 36 | – | – | – |
| | 1980–89 | – | M | 3445 | 23 (9.8) | 23 | – | – | – |
| | | – | M | 1200 | 25 (9.4) | – | – | 25 | – |

[a]With or without folic acid. [b]Per 100 ml.

leukaemia in general and acute lymphoblastic leukaemia in particular. Indeed, for the leukaemias, the only statistically significant associations were in the acute myeloid group, in which increased risks were found for caesarean section (OR ∞; 95% CI 1.2–∞) and for birthweights of more than 3500 g (OR 6.2; 95% CI 1.3–29.8). The finding for caesarean section is, however, based on only two cases, one of whom weighed 3925 g at birth.

In the acute myeloid group, the average birthweights were 3615 g (standard error 107 g) and 3215 g (s.e. 84 g) for cases and controls respectively. Further information about the relation between birthweight and leukaemia is presented in Table 7, which shows the age-specific data and also a comparison between index babies (cases and controls) and, for those whose mothers had a previous birth, their immediately preceding siblings. Overall, for age, the odds ratios tend to increase as age increases, but the trend is not statistically significant ($P$ = 0.06, for all leukaemias combined). The findings for birthweights of preceding siblings are perhaps more intriguing: at 7.6 (95% CI 0.9–63.9), the odds ratio for birthweights of 3500 g or more among preceding siblings in the acute myeloid group are of borderline statistical significance. In addition, there is some suggestion that the preceding siblings of children diagnosed with acute lymphoblastic leukaemia were heavier than the preceding siblings of their corresponding controls (OR 1.9; 95% CI 0.9–4.2), contributing to the fact that the odds ratio for all leukaemias combined was 2.2 (95% CI 1.1–4.4).

Information about maternal illnesses, radiography and drugs prescribed during pregnancy are presented in Table 8. Two notable case–control differences were found for maternal illnesses during pregnancy; the first being for viral infection and the second for anaemia. In the leukaemia group, information about a viral infection during pregnancy was recorded in the notes of six case mothers and two control mothers (OR 6.0; 95% CI 1.2–29.7) and, for non-Hodgkin's lymphoma, in the notes of two case mothers and no control mothers, yielding an odds ratio of infinity (95% CI 1.2–∞). Further information on the eight infections in the mothers of case children and the two infections in the mothers of control

© Cancer Research Campaign 1997

children are listed in Table 9. The mothers of both control children were diagnosed with influenza, as were three of the mothers whose children went on to develop acute lymphoblastic leukaemia. Of the remaining five cases, two of their mothers had vulval warts, one had herpes simplex, one had rubella and one had chicken pox. All eight case children were under 8 years old when their disease was diagnosed, and the gestational ages at in utero infection ranged from 6 to 34 weeks. Furthermore, although the numbers are small, it may be worth noting the sexes of the affected children: only two were female (both of whose mothers had genital warts), a male–female sex ratio of 3.0.

Seventeen mothers of leukaemic children and 15 mothers of control children (Table 8) were diagnosed with anaemia during the index pregnancy (OR 2.4; 95% CI 1.2–5.0). Of those so diagnosed, 11 cases and six controls had at least one haemoglobin below 10 g (OR 3.8; 95% CI 1.3–11.1). No association with non-Hodgkin's lymphoma and anaemia is evident. Further information about the 17 mothers and children in the leukaemic group (11 cases and six controls) with haemaglobins below 10 g is presented in Table 10. Details about therapy for anaemia were recorded in the notes of 9 (82%) of the 11 cases and all five of the controls: three case mothers and one control mother were transfused, and two of the transfused case mothers had a bone marrow biopsy. Despite their mothers' anaemia, the leukaemic case babies appeared remarkably healthy, with birthweights ranging from 3290 g to 4860 g (mean 3854 g). Two notable features of the leukaemic cases are the female preponderance (male–female sex ratio 0.8) and their comparatively late age at diagnosis: the odds ratios increase from 1.3 (95% CI 0.2–8.0, based on two cases and three controls) under 5 years of age to 3.9 (95% CI 0.7–20.9, based on five cases and three controls) at 5–14 years and reach infinity (based on four cases and no controls) at 15 years of age or more (test for trend with age; $Z = 3.6$, $P < 0.01$).

No other statistically significant associations were found for exposures during pregnancy, although the findings for anticonvulsant usage and pelvimetry are similar to those that have been reported before (Table 8). In addition to the data given in Table 8, information about a range of other drugs and vitamins were recorded in the hospital notes; all were examined and no statistically significant associations were found. The findings are not presented here because the numbers of subjects were often small, and there was considerable overlap between the exposures – some women having several drugs listed while the majority had none at all. In view of the findings for anaemia, however, it is notable that no case–control differences with respect to the prescription of iron and/or folic acid were apparent.

### DISCUSSION

The investigation described here was specifically designed to examine the relation between prenatal and neonatal factors and the subsequent development of haematological malignancies in children and young adults, its success depending on the ability to link routinely collected cancer registration data with good quality obstetric information. While the results presented here could have been affected by chance because of small numbers, we believe that they are unlikely to have been biased; information about exposure was abstracted from records compiled before diagnosis, and the 'find' rate for obstetric records was high at 85%. Inspection of the delivery records of case and control babies whose mother's obstetric notes could not be traced, revealed nothing unusual (data not shown), making it unlikely that the obstetric records of subjects whose notes we could not find differed in important respects from those we could. Further, although the four research nurses (three midwives and one paediatric nurse) who traced and abstracted the medical notes were not 'blind' to case–control status, great care was taken to ensure that such knowledge did not result in biased data collection; the tightly structured abstraction forms and coding procedures were designed and tested before the study began, and cross-checks in the form of duplicate abstractions and coding were used for initial training and periodically throughout for quality control.

The study does, however, have certain weaknesses, although it is not clear how our findings would have been influenced by them. Some individuals diagnosed with cancer who were born within the catchment areas of the study hospitals (or their predecessors) in the years for which obstetric data were being obtained will have been missed, either because their NHS number was not added to the cancer registration databases or because their cancer was not registered in the national scheme. Estimating this shortfall with any degree of accuracy is not possible as reliable cancer registration data are not available for the earlier years and annual tallies of births were not kept at the study hospitals. Further, while we know that the controls were alive and had no serious anomalies diagnosed before discharge and that they did not have a cancer registration with an NHS number attached, we cannot be certain that they were alive and cancer free when their corresponding case was diagnosed.

### Characteristics of the baby and neonatal exposures

There is strong evidence that certain genetic conditions predispose towards malignancy in later life, male sex and Down's syndrome being well-recognized risk factors for childhood leukaemia for example Doll (1989). In the present study, cases and controls were matched on sex, and babies with chromosomal anomalies were excluded as these variables would have acted as strong confounders in many of the analyses.

A number of investigators have reported that heavy birthweight is a risk factor for childhood leukaemia (MacMahon and Newill, 1962; Fasal et al, 1971; Wertelecki and Mantel, 1973; Shu et al, 1988; Kaye et al, 1991; Cnattingius et al, 1995; Ross et al, 1996), although others have found no such relation (McKinney et al, 1987; Golding et al, 1990; Zack et al, 1991). Most studies to date have concentrated either on infants or on children diagnosed before the age of 15 years, some concluding that the association between birthweight and leukaemia is strongest for younger ages, some that it is more pronounced for acute lymphoblastic leukaemia and some that the birthweight effect predominates in one sex or the other (for review see Ross et al, 1996).

On balance, our findings support the view that factors associated with fetal growth may also be associated with the subsequent development of leukaemia, particularly of the acute myeloid type in which a sixfold increase in risk was found for babies weighing more than 3500 g. However, no statistically significant trends with age at diagnosis were detected for all leukaemias combined or for acute lymphoblastic leukaemia alone, and examination of our data for men and women separately did not reveal any systematic differences (data not shown). Further, the observation that older siblings of leukaemic children were also comparatively heavy at birth cautions against ascribing a direct causal link between birthweight and leukaemia, the inference being that other factor(s) could be responsible for both phenomena.

© Cancer Research Campaign 1997

As well as having relatively high birthweights, the leukaemic cases appeared comparatively robust at birth with respect to other indicators of well-being, the odds ratios for jaundice, phototherapy, admission to special care nursery and neonatal intensive care all being less than 1.0. Although the numbers are small, it should be noted that our findings for phototherapy agree with those of Cnattigius and colleagues (1995), offering little support for the hypothesis that neonatal exposure to strong illumination is a material cause of acute lymphoblastic leukaemia (Ben-Sasson and Davis, 1992).

The lack of an association between leukaemia and administration of intramuscular vitamin K to the neonate also deserves particular attention as, after the report of Golding and colleagues in 1992, this issue has been the subject of considerable debate (Draper and Stiller, 1992; Ekelund et al, 1993; Olsen et al, 1994; Ansell et al, 1996; von Kreis et al, 1996; Zipursky et al, 1996). The retrospective assessment of whether a baby received vitamin K and by what route is not straightforward (Ansell et al, 1996) and, because of this, we presented our results in two ways, firstly by what was recorded in the notes and secondly by what could be imputed about hospital policy. With either method, our findings do not support the suggestion of an association between intramuscular vitamin K and leukaemia. In a recent report von Kreis and colleagues (1996) presented data on acute lymphoblastic leukaemia for children aged between the ages of one and six years calculating an adjusted odds ratio for administration of intramuscular vitamin K of 1.2 (95% CI 0.7–2.2). For comparative purposes, our data for the same disease group and age range produced an odds ratio of 0.6 (95% CI 0.3–1.4) based on hospital notes and 0.6 (95% CI 0.2–1.7) based on hospital policy; adjustment for potential confounders, such as admission to a special care nursery and mode of delivery, had no material effect.

Relatively little obstetric data has been published for non-Hodgkin's lymphoma, and the present report only contains information on 34 cases. McKinney and colleagues (1987) studied 31 children with non-Hodgkin's lymphoma and found that they were significantly lighter at birth than their corresponding controls. As we also found that at birth the non-Hodgkin's lymphoma cases appeared generally disadvantaged (although not significantly so) by comparison with their own controls, and by comparison with the leukaemic cases, further research on the relation between prenatal and neonatal factors and non-Hodgkin's lymphoma may be warranted.

### In utero exposures

The suggestion that in utero exposure to viral infection, particularly influenza and varicella, may predispose towards leukaemia is not new, although the evidence is inconsistent (Stewart et al, 1958; Adelstein and Donovan, 1972; Fedrick and Alberman, 1972; Leck and Steward, 1972; Doll, 1973; Hakulinen et al, 1973; Fine et al, 1985; McKinney et al, 1987; Gilman et al, 1989; Anon, 1990; Ross et al, 1994). Taken at face value, the approximately sixfold increased risk found here supports the hypothesis that prenatal viral infection may be related to the subsequent development of not only childhood leukaemia (six cases and two controls) but also of childhood non-Hodgkin's lymphoma (two cases and no controls). However, as with birthweight, this finding should not necessarily be taken to imply a direct causal relation as the documented exposures relate to the manifestation of clinically diagnosed maternal viral disease and not to documented fetal exposure to a viral infection. Unfortunately, information about whether the two cases of vulval warts and the one case of herpes simplex were incident cases (diagnosed for the first time in the index pregnancy) or were severe eruptions of a pre-existing condition were not recorded in the notes.

As far as we are aware, the possibility that maternal anaemia in pregnancy may be related to leukaemia has not been suggested before, although data contained within a report on the Oxford Survey of Childhood Cancers show a positive association between anaemia and all cancers combined (Gilman et al, 1989). A striking feature of our data is the difference between the ages and sexes of the leukaemias associated with anaemia in pregnancy and those associated with viral infection: the former being predominantly older female cases and the latter younger male cases. As with the findings for birthweight and viral infection, however, the meaning of the almost fourfold increase in risk associated with a maternal haemoglobin below 10 g is unclear. Although it is possible that lack of a nutrient while in utero, such as iron or folate, could predispose towards leukaemia, it is also possible that the mother's anaemia and the offspring's leukaemia could, in fact, share a common cause.

Apart from viral infection and anaemia, there was little support within our data for associations between leukaemia and maternal use of anticonvulsants or antibiotics during pregnancy or with drugs given to the mother in labour, the odds ratios for anaesthetics, entonox and pethidine all being close to unity (Doll, 1973; Kinnier-Wilson et al, 1981; McKinney et al, 1985; Robison et al, 1988; Gilman et al, 1989; Golding et al, 1990; Cnattingius et al, 1995).

### Maternal characteristics and birth order

A number of investigators have suggested that certain characteristics of the mother could predispose towards leukaemia in her offspring. This is a complex area as disentangling relationships between a mother's prior reproductive history, her age and the birth order of the affected child is not straightforward.

Although some researchers have found an association with advanced maternal age (Stewart et al, 1958; MacMahon and Newill, 1962; Stark and Mantel, 1966; Shaw et al, 1984; Kaye et al, 1991), others have not (Fasal et al, 1971; Salonen, 1976; van Steensel-Moll et al, 1985; McKinney et al, 1987; Shu et al, 1988; Golding et al, 1990, 1992; Kaye et al, 1991; Zack et al, 1991; Cnattingius et al, 1995). Similarly, while it has been suggested that being the first-born child may be a risk factor for leukaemia (MacMahon and Newill, 1962; Stark and Mantel, 1966; van Steensel-Moll et al, 1986; MacMahon, 1992), many studies have failed to confirm this association (Fasal et al, 1971; Salonen, 1976; Shaw et 1984; McKinney et al, 1987; Shu et al, 1988; Golding et al, 1990, 1992; Kaye et al, 1991; Zack 1991; Zack et al, 1991; Roman et al, 1994; Cnattingius et al, 1995). Likewise, although it has been hypothesized that mothers of leukaemic children are more likely to have had prior fetal deaths and have fertility problems (van Steensel-Moll et al, 1985), the evidence is sparse and contradictory (MacMahon and Newill, 1962; Shu et al, 1988; Kaye et al, 1991; Cnattingius et al, 1995)

We found no evidence of any link between leukaemia and birth order, but our findings with respect to maternal age, numbers of previous pregnancies and fertility treatment highlight the complexity of the issues – the mothers of leukaemic cases being marginally older, having slightly fewer past pregnancies and being more likely to have had fertility treatment than their corresponding

© Cancer Research Campaign 1997

controls. None of the differences were statistically significant, but in view of current trends with respect to fertility treatment this area may warrant further research.

### CONCLUSION

The findings presented here contribute to the accumulating body of knowledge about possible prenatal origins of haematological cancers. The methods and procedures used proved to be reliable, and the investigation has now been extended to include other malignancies and other hospitals.

While some hypotheses were supported by our analyses (e.g. in utero viral exposure), others were not (e.g. neonatal vitamin K). In addition, novel associations that require confirmation in future research have emerged (e.g. maternal anaemia in pregnancy). Information on larger numbers of cases incorporating more refined diagnostic and biological information are clearly required. For children diagnosed before the age of 15 years, this should be provided by the United Kingdom Childhood Cancer Study (UKCCS), which is a collaborative study of several thousand children diagnosed with cancer in the UK. Our findings suggest, however, that prenatal factors may be important determinants of haematological malignancies in young adults, as well as in children. Given that the clearest example to date of an in utero exposure causing cancer is that of maternal diethylstilboestrol use in pregnancy and clear cell adenocarcinoma in young women (Herbst et al, 1971), and considering the suggestions that testis cancer (Swerdlow et al, 1987) and breast cancer (Ekbom et al, 1992; Michels et al, 1996) may have a prenatal component to their aetiology, this is perhaps not unexpected.

### ACKNOWLEDGEMENTS

We thank Gerald Draper, Charles Stiller and Tony Swerdlow for advice and help with case ascertainment; Judith Black, Susie Boon and Pat Townshend for data collection; the hospital staff who helped trace the records; and Valerie Beral, Ray Cartwright, Richard Doll, Pat Doyle and Patricia McKinney for comments on a previous draft. The study was funded by the Imperial Cancer Research Fund.

### REFERENCES

Adelstein AM and Donovan JW (1972) Malignant disease in children whose mothers had chickenpox, mumps or Rubella in pregnancy. *Br Med J* **4**: 629–631

Anon (1990) Childhood leukaemia: an infectious disease? *Lancet* **336**: 1477–1479

Ansell P, Bull D and Roman E (1996) Childhood leukaemia and intramuscular vitamin K: findings from a case-control study. *Br Med J* **313**: 204

Ben-Sasson SA and Davis DL (1992) Neonatal exposure to protoporphyrin-activating lighting as a contributing cause of childhood acute lymphocytic leukaemia. *Cancer Causes Control* **3**: 383–387

Bithell JF and Stiller CA (1988) A new calculation of the carcinogenic risk of obstetric x-raying. *Statist Med* **7**: 857–864

Breslow NE and Day NE (1980) *Statistical Methods in Cancer Research.* Vol I. *The Analysis of Case-Control Studies.* International Agency for Research on Cancer (IARC) Scientific Publications: Lyon

Cnattingius S, Zack MM, Ekbom A, Gunnarskog J, Kreuger A, Linet M and Adami H-O (1995) Prenatal and neonatal risk factors for childhood lymphatic leukaemia. *J Natl Cancer Inst* **87**: 908–914

Doll R (1973) Hazards of the first nine months: an epidemiologists nightmare. *J Irish Med Assoc* **66**: 117–126

Doll R (1989) The epidemiology of childhood leukaemia. *J R Statist Soc A* **152**: 341–351

Draper GJ and Stiller CA (1992) Intramuscular vitamin K and childhood cancer. *Br Med J* **305**: 709

Ekbom A, Trichopolous D, Adami H-O, Hsieh C and Lan S (1992) Evidence of prenatal influences on breast cancer risk. *Lancet* **340**: 1015–1018

Ekelund H, Finnstrom O, Gunnatskog J, Kallen E and Larsson Y (1993) Administration of vitamin K to newborn infants and childhood cancer. *Br Med J* **301**: 89–91

Fasal E, Jackson EW and Klauber MR (1971) Birth characteristics and leukaemia in childhood. *J Natl Cancer Inst* **47**: 501–509

Fedrick J and Alberman ED (1972) Reported influenza in pregnancy and subsequent cancer in the child. *Br Med J* **2**: 485–488

Fine PEM, Adelstein AM, Snowman J, Clarkson JA and Evans SM (1985) Long term effects of exposures to viral infections in utero. *Br Med J* **290**: 509–511

Ford AM, Ridge SA, Cabrera ME, Mahmoud H, Steel CM, Chan LC and Greaves M (1993) In utero rearrangements in the trithorax-related oncogene in infant leukaemias. *Nature* **363**: 358–360

Ford AM, Pombo-de-Oliveria, McCarthy KP, Maclean JM, Carrico KC, Vincent RF and Greaves M (1997) Monoclonal origin of concordant T-cell malignancy in identical twins. *Blood* **89**: 281–285

Gilman EA, Kneale GW, Knox EG and Stewart AM (1988) Pregnancy X-rays and childhood cancers: effects of exposure age and radiation dose. *J Radiol Prot* **8**: 3–8

Gilman EA, Wilson LMK, Kneale GW and Waterhouse JAH (1989) Childhood cancers and their association with pregnancy drugs and illnesses. *Paediat Perinatal Epidemiol* **3**: 66–94

Golding J, Paterson M and Kinlen LJ (1990) Factors associated with childhood cancer in a national cohort study. *Br J Cancer* **62**: 304–308

Golding J, Greenwood R, Birmingham K and Mott M (1992) Childhood cancer, intramuscular vitamin K, and pethidine given during labour. *Br Med J* **305**: 341–346

Hakulinen T, Hovi L, Karkinen-Jaaskelainen M, Penttinen K and Saxen L (1973) Association between influenza during pregnancy and childhood leukaemia. *Br Med J* **4**: 265–267

Herbst A, Ulfelder H and Poskanzer DC (1971) Adenocarcinoma of the vagina. Association of maternal Stilbestrol therapy with tumor appearance in young women. *N Engl J Med* **284**: 878–881

Kaye SA, Robison LL, Smithson WA, Gunderson P, King FL and Neglia JP (1991) Maternal reproductive history and birth characteristics in childhood acute lymphoblastic leukaemia. *Cancer* **68**: 1351–1355

Kinnier-Wilson NM, Kneale GW and Stewart AM (1981) Childhood cancer and pregnancy drugs. *Lancet* **ii**: 314–315

Leck I and Steward JK (1972) Incidence of neoplasms in children born after influenza epidemics. *Br Med J* **4**: 631–634

MacMahon B (1992) Is acute lymphoblastic leukaemia in children virus-related? *Am J Epidemiol* **136**: 916–914

MacMahon B and Newill VA (1962) Birth characteristics of children dying of malignant neoplasms. *J Natl Cancer Inst* **28**: 231–244

McKinney PA, Cartwright RA, Stiller CA, Hopton PA, Mann JR, Birch JM, Hartley AL, Waterhouse JAH and Johnston HE (1985) Inter-regional epidemiological study of childhood cancer (IRESCC): childhood cancer and the consumption of Debendox and related drugs in pregnancy. *Br J Cancer* **52**: 923–929

McKinney PA, Cartwright RA, Saiu JMT, Mann JR, Stiller CA, Draper GJ, Hartley AL, Hopton PA, Birch JM, Waterhouse JAH and Johnston HE (1987) The inter-regional epidemiological study of childhood cancer (IRESCC): a case–control study of aetiological factors in leukaemia and lymphoma. *Arch Dis Child* **62**: 279–287

Michels KB, Trichopolous D, Robins JM, Rosner BA, Manson JE, Hunter DJ, Colditz GA, Hankinson SE, Speizer FE and Willett WC (1996) Birthweight as a risk factor for breast cancer. *Lancet* **348**: 1542–1546

Mole RH (1990) Childhood cancer after prenatal exposure to diagnostic X-ray examinations in Britain. *Br J Cancer* **62**: 152–168

Olsen JH, Hertz H, Blinkenberg K and Verder H (1994) Vitamin K regimes and incidence of childhood cancer in Denmark. *Br Med J* **301**: 89–91

Preston DL, Lubin JH and Pierce DA (1993) *Epicure Users Guide.* Mirosoft International: Seattle, WA

Robison LL, Buckley JD, Daigle AE, Wells R, Benjamin D, Arthur DC and Hammond GD (1988) Maternal drug use and risk of childhood non-lymphoblastic leukaemia among offspring. *Cancer* **63**: 1904–1911

Roman E, Watson A, Bull D and Baker K (1994) Leukaemia risk and social contact in children aged 0–4 years in Southern England. *J Epidemiol Commun Hlth* **48**: 601–602

Ross JA, Davies SM, Potter JD and Robison LL (1994) Epidemiology of childhood leukaemia, with a focus on infants. *Epidemiol Rev* **16**: 243–271

© Cancer Research Campaign 1997

Ross JA, Perentesis JP, Robison LL and Davies SM (1996) Big babies and infant leukaemia: a role for insulin-like growth factor-1? *Cancer Causes Control* **7**: 553–569

Salonen T (1976) Prenatal and perinatal factors in childhood cancer. *Ann Clin Res* **7**: 27–42

Shaw G, Lavey R, Jackson R and Austin D (1984) Association of childhood cancer with maternal age, birth order and paternal occupation. *Am J Epidemiol* **119**: 788–795

Shu XO, Gao YT, Brinton LA, Linet MS, Tu JT, Zheng W and Fraumeni JF (1988) A population-based case–control study of childhood leukaemia in Shanghai. *Cancer* **62**: 635–644

SPSS (1989) *Statistical Package for the Social Sciences -X: Users Guide*. McGraw Hill: Chicago

Stark CR and Mantel N (1966) Effects of maternal age and birth order on the risks of mongolism and leukaemia. *J Natl Cancer Inst* **37**: 687–698

Stata (1995) *Stata Statistical Software: Release 4.0.* Stata Corporation: College Station, Texas.

Stewart A, Webb J, Giles D and Hewitt D (1956) Malignant disease in childhood and diagnostic irradiation in utero. *Lancet* **2**: 447

Stewart A, Webb J and Hewitt D (1958) A survey of childhood malignancies. *Br Med J* **28**: 1495–1507

Stiller CA, Allen MB and Eatock EM (1995) Childhood cancer in Britain: the National Registry of Childhood Tumours and Incidence Rates 1978–1987. *Eur J Cancer* **31A**: 2028–2034

Swerdlow AJ, Huttly SRA and Smith PG (1987) Prenatal and familial associations of testicular cancer. *Br J Cancer* **55**: 571–577

Van Steensel-Moll HA, Valkenburg HA, Vandenbroucke JP and Van Zanen GE (1985) Are maternal fertility problems related to childhood leukaemia? *Int J Epidemiol* **14**: 555–559

Van Steensel-Moll HA, Valkenberg HA and Van Zanen GE (1986) Childhood leukaemia and infectious diseases in the first year of life: a register-based case–control study. *Am J Epidemiol* **124**: 590–594

Von Kries R, Gobel U, Hachmeister A, Kaletsch U and Michaelis J (1996) Vitamin K and childhood cancer: a population based case–control study in Lower Saxony, Germany. *Br Med J* **313**: 199–203

Wakeford R (1995) The risk of childhood cancer from intrauterine and preconceptional exposure to ionizing radiation. *Environ Hlth Perspec* **103**: 1018–1025

Wertelecki W and Mantel N (1973) Increased birth weight in leukaemia. *Paediat Res* **7**: 132–138

Zack M, Adami H-O and Ericson A (1991) Maternal and perinatal risk factors for childhood leukaemia. *Cancer Res* **51**: 3696–3701

Zipursky A (1996) Vitamin K at birth. *Br Med J* **313**: 179–180

© Cancer Research Campaign 1997